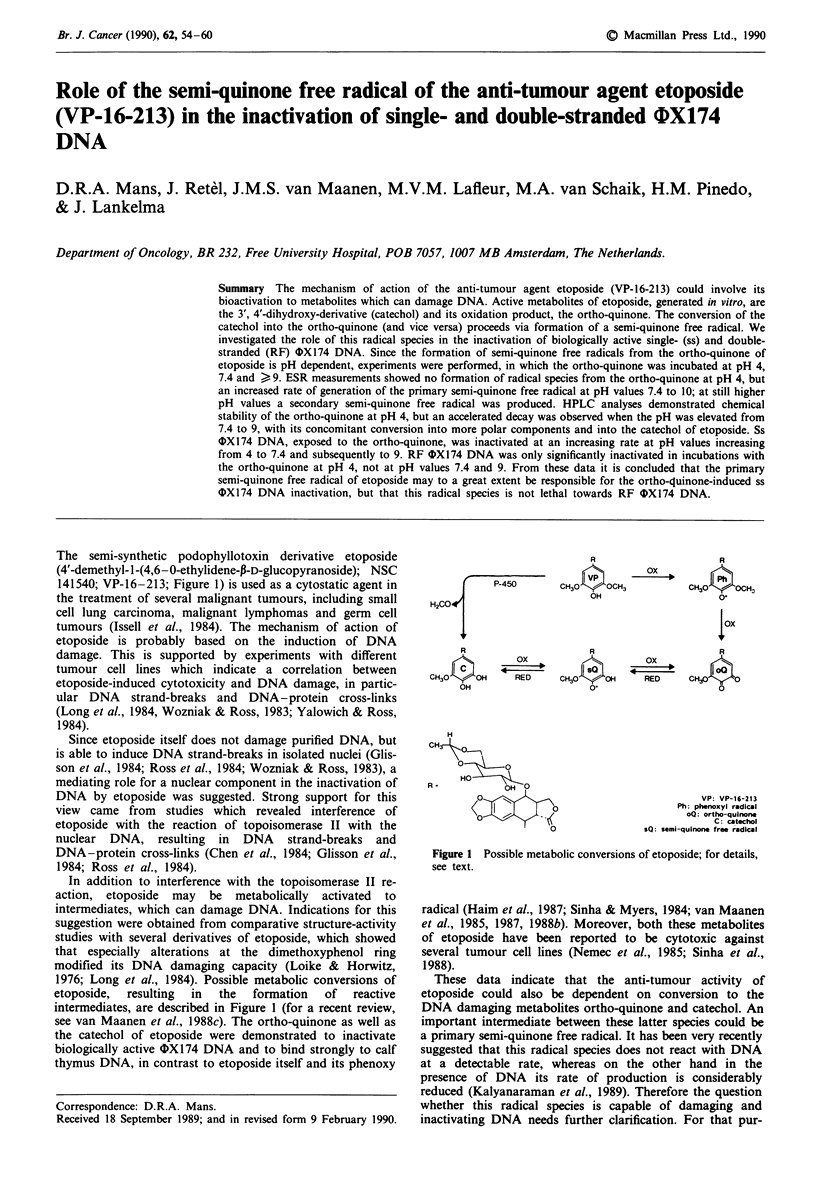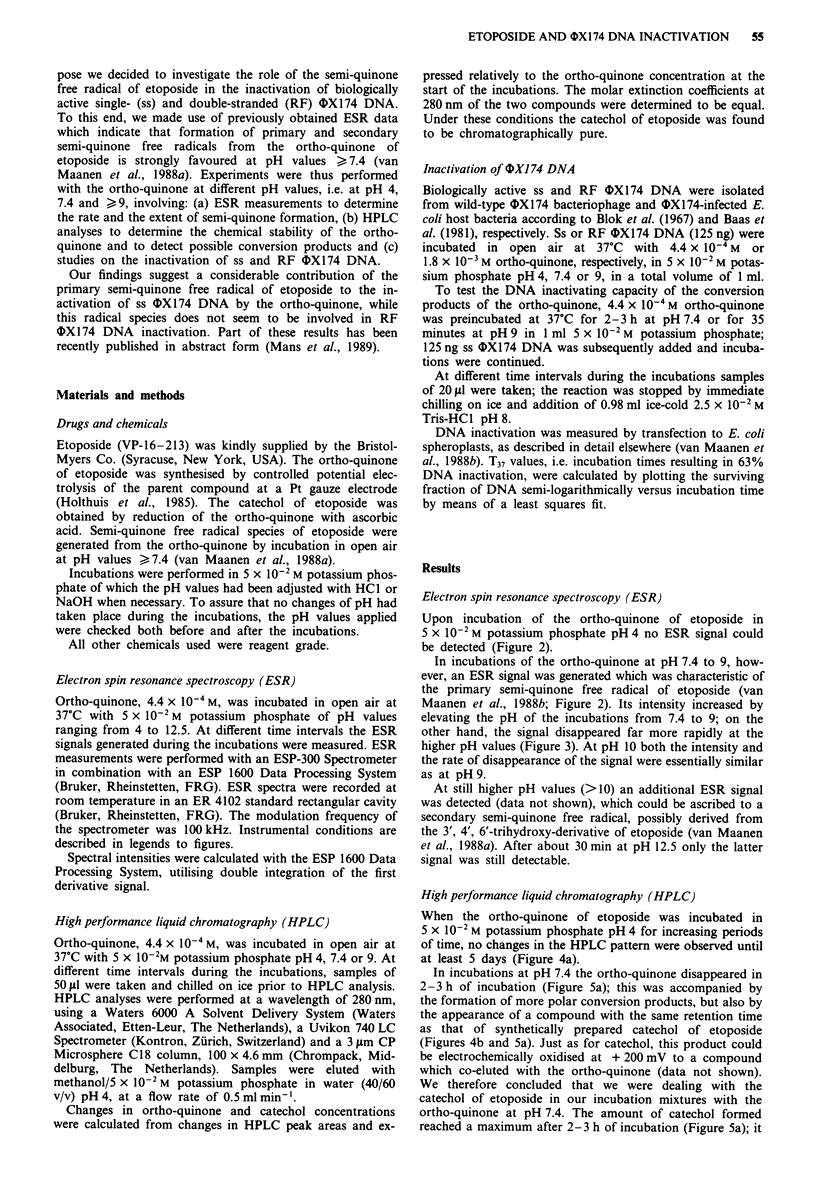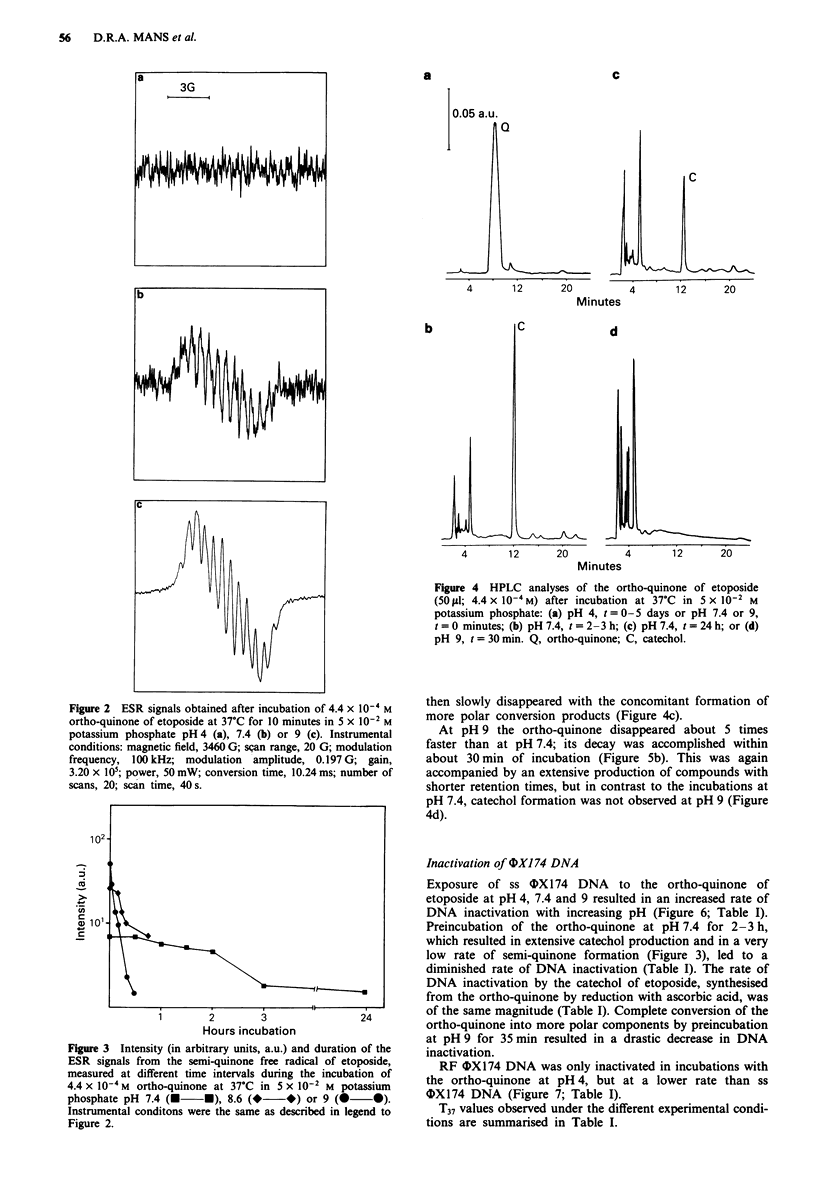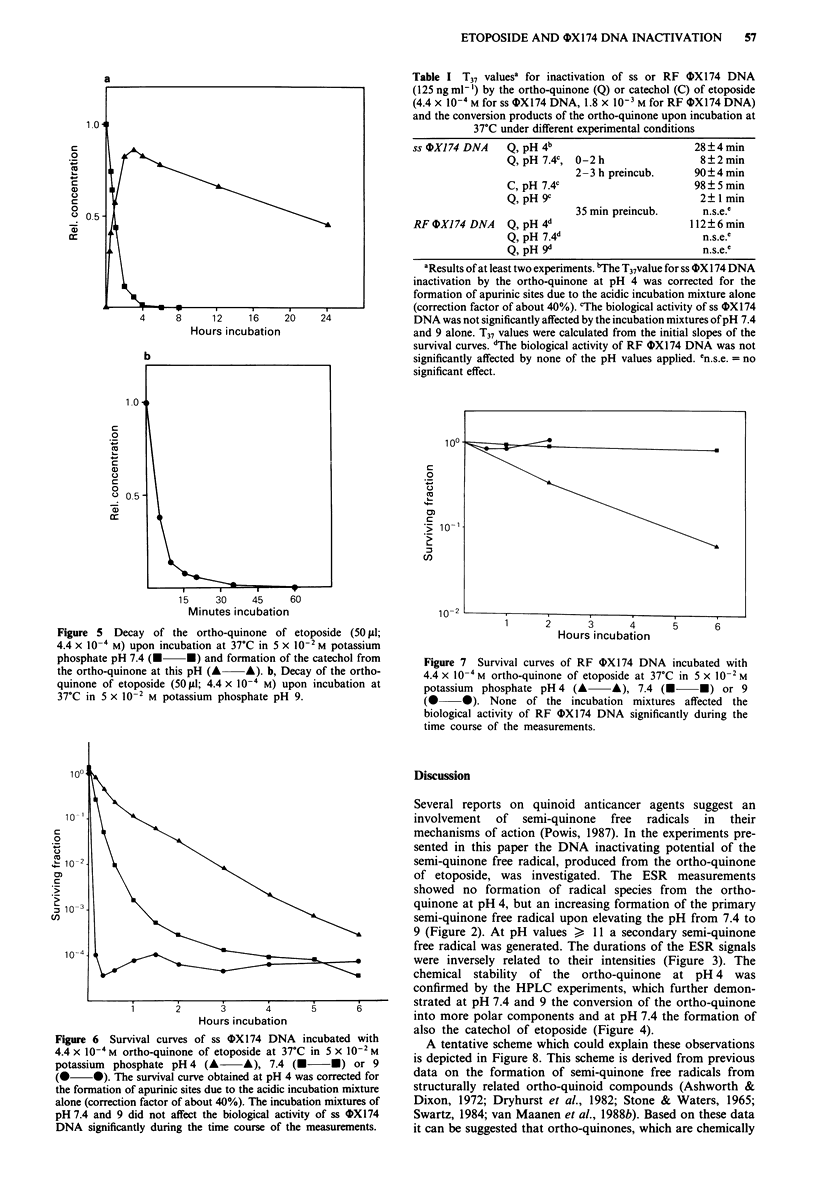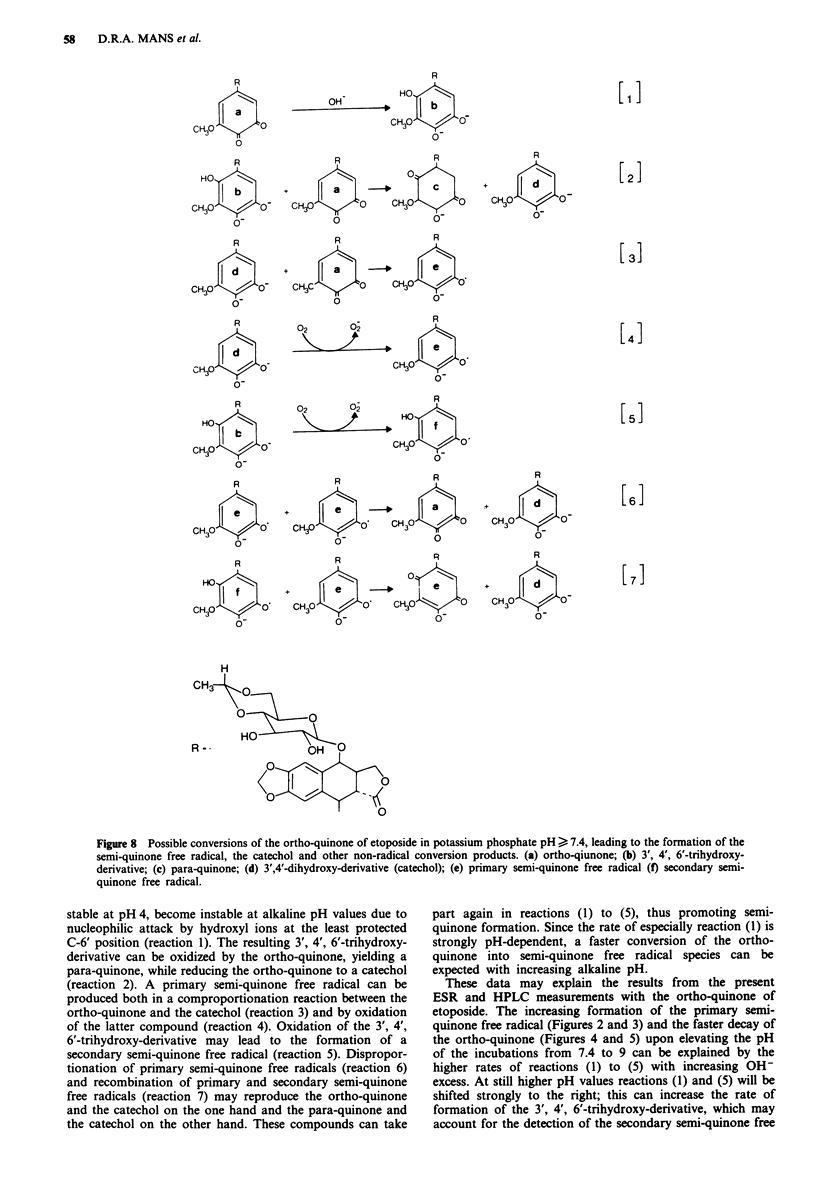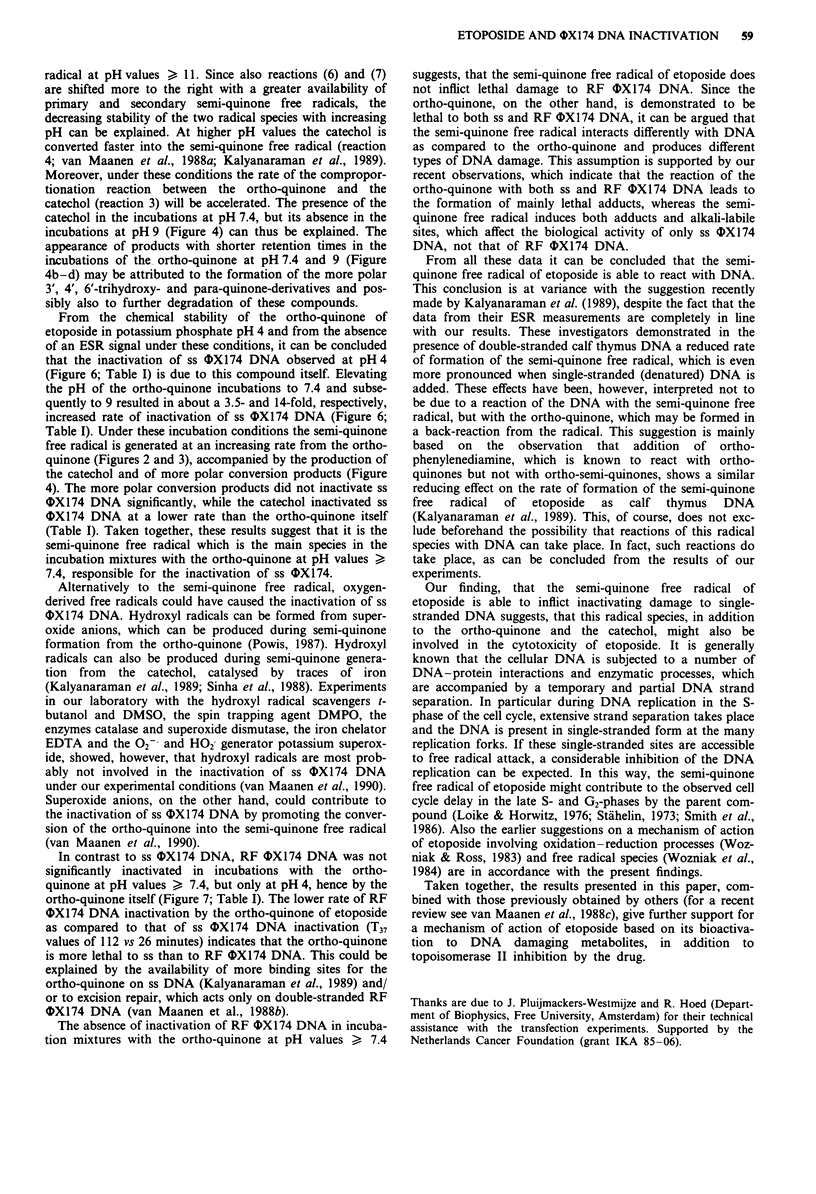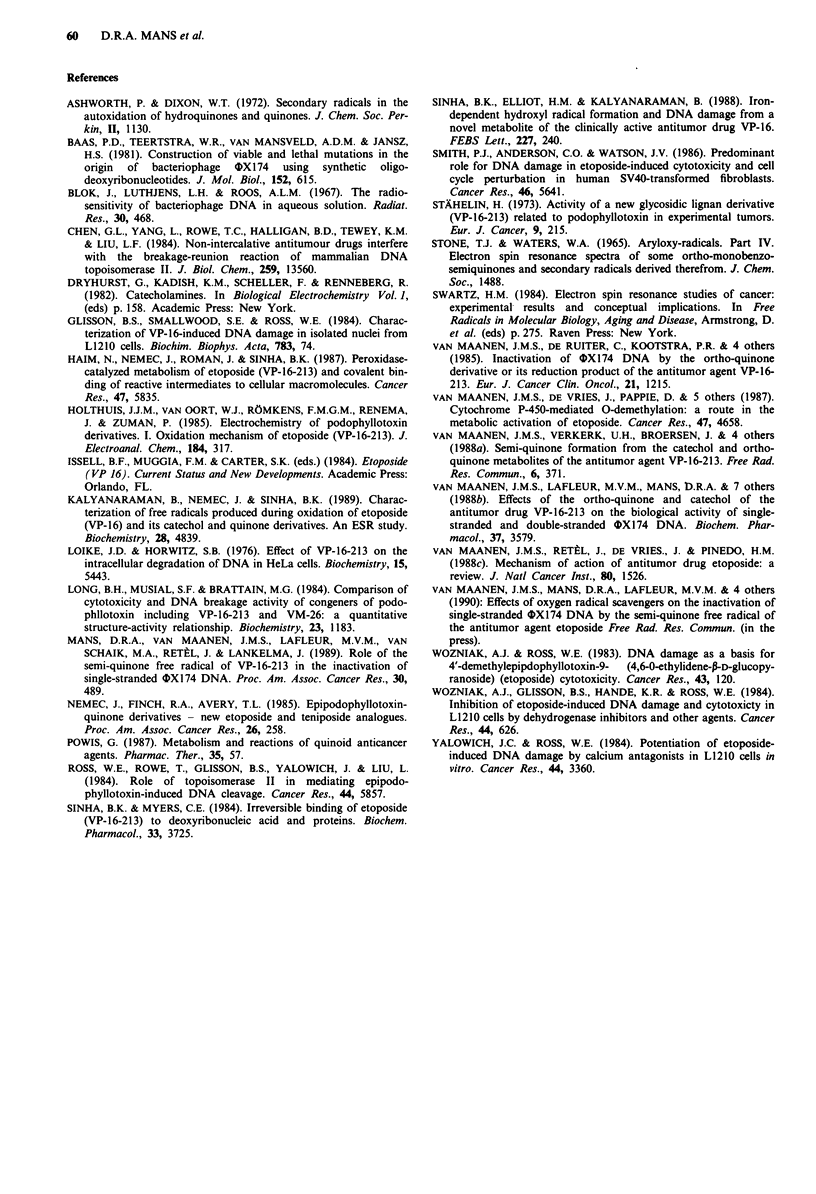# Role of the semi-quinone free radical of the anti-tumour agent etoposide (VP-16-213) in the inactivation of single- and double-stranded phi X174 DNA.

**DOI:** 10.1038/bjc.1990.228

**Published:** 1990-07

**Authors:** D. R. Mans, J. Retèl, J. M. van Maanen, M. V. Lafleur, M. A. van Schaik, H. M. Pinedo, J. Lankelma

**Affiliations:** Department of Oncology, Free University Hospital, Amsterdam, The Netherlands.

## Abstract

The mechanism of action of the anti-tumour agent etoposide (VP-16-213) could involve its bioactivation to metabolites which can damage DNA. Active metabolites of etoposide, generated in vitro, are the 3',4'-dihydroxy-derivative (catechol) and its oxidation product, the ortho-quinone. The conversion of the catechol into the ortho-quinone (and vice versa) proceeds via formation of a semi-quinone free radical. We investigated the role of this radical species in the inactivation of biologically active single- (ss) and double-stranded (RF) phi X174 DNA. Since the formation of semi-quinone free radicals from the ortho-quinone of etoposide is pH dependent, experiments were performed, in which the ortho-quinone was incubated at pH 4, 7.4 and greater than or equal to 9. ESR measurements showed no formation of radical species from the ortho-quinone at pH 4, but an increased rate of generation of the primary semi-quinone free radical at pH values 7.4 to 10; at still higher pH values a secondary semi-quinone free radical was produced. HPLC analyses demonstrated chemical stability of the ortho-quinone at pH 4, but an accelerated decay was observed when the pH was elevated from 7.4 to 9, with its concomitant conversion into more polar components and into the catechol of etoposide. Ss phi X174 DNA, exposed to the ortho-quinone, was inactivated at an increasing rate at pH values increasing from 4 to 7.4 and subsequently to 9. RF phi X174 DNA was only significantly inactivated in incubations with the ortho-quinone at pH 4, not at pH values 7.4 and 9. From these data it is concluded that the primary semi-quinone free radical of etoposide may to a great extent be responsible for the ortho-quinone-induced ss phi X174 DNA inactivation, but that this radical species is not lethal towards RF phi X174 DNA.


Br. J. Cancer (1990), 62, 54–60 © Macmillan Press Ltd., 1990

### Role of the semi-quinone free radical of the anti-tumour agent etoposide (VP-16-213) in the inactivation of single- and double-stranded ΦX174 DNA

D.R.A. Mans, J. Retèl, J.M.S. van Maanen, M.V.M. Lafleur, M.A. van Schaik, H.M. Pinedo, & J. Lankelma

*Department of Oncology, BR 232, Free University Hospital, POB 7057, 1007 MB Amsterdam, The Netherlands.*

**Summary** The mechanism of action of the anti-tumour agent etoposide (VP-16-213) could involve its bioactivation to metabolites which can damage DNA. Active metabolites of etoposide, generated *in vitro*, are the 3', 4'-dihydroxy-derivative (catechol) and its oxidation product, the ortho-quinone. The conversion of the catechol into the ortho-quinone (and vice versa) proceeds via formation of a semi-quinone free radical. We investigated the role of this radical species in the inactivation of biologically active single- (ss) and double-stranded (RF) ΦX174 DNA. Since the formation of semi-quinone free radicals from the ortho-quinone of etoposide is pH dependent, experiments were performed, in which the ortho-quinone was incubated at pH 4, 7.4 and ⩾9. ESR measurements showed no formation of radical species from the ortho-quinone at pH 4, but an increased rate of generation of the primary semi-quinone free radical at pH values 7.4 to 10; at still higher pH values a secondary semi-quinone free radical was produced. HPLC analyses demonstrated chemical stability of the ortho-quinone at pH 4, but an accelerated decay was observed when the pH was elevated from 7.4 to 9, with its concomitant conversion into more polar components and into the catechol of etoposide. Ss ΦX174 DNA, exposed to the ortho-quinone, was inactivated at an increasing rate at pH values increasing from 4 to 7.4 and subsequently to 9. RF ΦX174 DNA was only significantly inactivated in incubations with the ortho-quinone at pH 4, not at pH values 7.4 and 9. From these data it is concluded that the primary semi-quinone free radical of etoposide may to a great extent be responsible for the ortho-quinone-induced ss ΦX174 DNA inactivation, but that this radical species is not lethal towards RF ΦX174 DNA.

The semi-synthetic podophyllotoxin derivative etoposide (4'-demethyl-1-(4,6-0-ethylidene-β-D-glucopyranoside); NSC 141540; VP-16-213; Figure 1) is used as a cytostatic agent in the treatment of several malignant tumours, including small cell lung carcinoma, malignant lymphomas and germ cell tumours (Issell *et al.*, 1984). The mechanism of action of etoposide is probably based on the induction of DNA damage. This is supported by experiments with different tumour cell lines which indicate a correlation between etoposide-induced cytotoxicity and DNA damage, in particular DNA strand-breaks and DNA–protein cross-links (Long *et al.*, 1984, Wozniak & Ross, 1983; Yalowich & Ross, 1984).

Since etoposide itself does not damage purified DNA, but is able to induce DNA strand-breaks in isolated nuclei (Glisson *et al.*, 1984; Ross *et al.*, 1984; Wozniak & Ross, 1983), a mediating role for a nuclear component in the inactivation of DNA by etoposide was suggested. Strong support for this view came from studies which revealed interference of etoposide with the reaction of topoisomerase II with the nuclear DNA, resulting in DNA strand-breaks and DNA–protein cross-links (Chen *et al.*, 1984; Glisson *et al.*, 1984; Ross *et al.*, 1984).

In addition to interference with the topoisomerase II reaction, etoposide may be metabolically activated to intermediates, which can damage DNA. Indications for this suggestion were obtained from comparative structure-activity studies with several derivatives of etoposide, which showed that especially alterations at the dimethoxyphenol ring modified its DNA damaging capacity (Loike & Horwitz, 1976; Long *et al.*, 1984). Possible metabolic conversions of etoposide, resulting in the formation of reactive intermediates, are described in Figure 1 (for a recent review, see van Maanen *et al.*, 1988*c*). The ortho-quinone as well as the catechol of etoposide were demonstrated to inactivate biologically active ΦX174 DNA and to bind strongly to calf thymus DNA, in contrast to etoposide itself and its phenoxy

**Figure 1** Possible metabolic conversions of etoposide; for details, see text.

radical (Haim *et al.*, 1987; Sinha & Myers, 1984; van Maanen *et al.*, 1985, 1987, 1988*b*). Moreover, both these metabolites of etoposide have been reported to be cytotoxic against several tumour cell lines (Nemec *et al.*, 1985; Sinha *et al.*, 1988).

These data indicate that the anti-tumour activity of etoposide could also be dependent on conversion to the DNA damaging metabolites ortho-quinone and catechol. An important intermediate between these latter species could be a primary semi-quinone free radical. It has been very recently suggested that this radical species does not react with DNA at a detectable rate, whereas on the other hand in the presence of DNA its rate of production is considerably reduced (Kalyanaraman *et al.*, 1989). Therefore the question whether this radical species is capable of damaging and inactivating DNA needs further clarification. For that pur-

Correspondence: D.R.A. Mans.
Received 18 September 1989; and in revised form 9 February 1990.

pose we decided to investigate the role of the semi-quinone free radical of etoposide in the inactivation of biologically active single- (ss) and double-stranded (RF) ΦX174 DNA. To this end, we made use of previously obtained ESR data which indicate that formation of primary and secondary semi-quinone free radicals from the ortho-quinone of etoposide is strongly favoured at pH values ⩾7.4 (van Maanen *et al.*, 1988*a*). Experiments were thus performed with the ortho-quinone at different pH values, i.e. at pH 4, 7.4 and ⩾9, involving: (a) ESR measurements to determine the rate and the extent of semi-quinone formation, (b) HPLC analyses to determine the chemical stability of the ortho-quinone and to detect possible conversion products and (c) studies on the inactivation of ss and RF ΦX174 DNA.

Our findings suggest a considerable contribution of the primary semi-quinone free radical of etoposide to the inactivation of ss ΦX174 DNA by the ortho-quinone, while this radical species does not seem to be involved in RF ΦX174 DNA inactivation. Part of these results has been recently published in abstract form (Mans *et al.*, 1989).

### Materials and methods

### *Drugs and chemicals*

Etoposide (VP-16–213) was kindly supplied by the Bristol-Myers Co. (Syracuse, New York, USA). The ortho-quinone of etoposide was synthesised by controlled potential electrolysis of the parent compound at a Pt gauze electrode (Holthuis *et al.*, 1985). The catechol of etoposide was obtained by reduction of the ortho-quinone with ascorbic acid. Semi-quinone free radical species of etoposide were generated from the ortho-quinone by incubation in open air at pH values ⩾7.4 (van Maanen *et al.*, 1988*a*).

Incubations were performed in $5 \times 10^{-2}$ M potassium phosphate of which the pH values had been adjusted with HCl or NaOH when necessary. To assure that no changes of pH had taken place during the incubations, the pH values applied were checked both before and after the incubations.

All other chemicals used were reagent grade.

### *Electron spin resonance spectroscopy (ESR)*

Ortho-quinone, $4.4 \times 10^{-4}$ M, was incubated in open air at 37°C with $5 \times 10^{-2}$ M potassium phosphate of pH values ranging from 4 to 12.5. At different time intervals the ESR signals generated during the incubations were measured. ESR measurements were performed with an ESP-300 Spectrometer in combination with an ESP 1600 Data Processing System (Bruker, Rheinstetten, FRG). ESR spectra were recorded at room temperature in an ER 4102 standard rectangular cavity (Bruker, Rheinstetten, FRG). The modulation frequency of the spectrometer was 100 kHz. Instrumental conditions are described in legends to figures.

Spectral intensities were calculated with the ESP 1600 Data Processing System, utilising double integration of the first derivative signal.

### *High performance liquid chromatography (HPLC)*

Ortho-quinone, $4.4 \times 10^{-4}$ M, was incubated in open air at 37°C with $5 \times 10^{-2}$M potassium phosphate pH 4, 7.4 or 9. At different time intervals during the incubations, samples of 50 µl were taken and chilled on ice prior to HPLC analysis. HPLC analyses were performed at a wavelength of 280 nm, using a Waters 6000 A Solvent Delivery System (Waters Associated, Etten-Leur, The Netherlands), a Uvikon 740 LC Spectrometer (Kontron, Zürich, Switzerland) and a 3 µm CP Microsphere C18 column, 100 × 4.6 mm (Chrompack, Middelburg, The Netherlands). Samples were eluted with methanol/$5 \times 10^{-2}$ M potassium phosphate in water (40/60 v/v) pH 4, at a flow rate of 0.5 ml $min^{-1}$.

Changes in ortho-quinone and catechol concentrations were calculated from changes in HPLC peak areas and expressed relatively to the ortho-quinone concentration at the start of the incubations. The molar extinction coefficients at 280 nm of the two compounds were determined to be equal. Under these conditions the catechol of etoposide was found to be chromatographically pure.

### *Inactivation of ΦX174 DNA*

Biologically active ss and RF ΦX174 DNA were isolated from wild-type ΦX174 bacteriophage and ΦX174-infected *E. coli* host bacteria according to Blok *et al.* (1967) and Baas *et al.* (1981), respectively. Ss or RF ΦX174 DNA (125 ng) were incubated in open air at 37°C with $4.4 \times 10^{-4}$ M or $1.8 \times 10^{-3}$ M ortho-quinone, respectively, in $5 \times 10^{-2}$ M potassium phosphate pH 4, 7.4 or 9, in a total volume of 1 ml.

To test the DNA inactivating capacity of the conversion products of the ortho-quinone, $4.4 \times 10^{-4}$ M ortho-quinone was preincubated at 37°C for 2–3 h at pH 7.4 or for 35 minutes at pH 9 in 1 ml $5 \times 10^{-2}$ M potassium phosphate; 125 ng ss ΦX174 DNA was subsequently added and incubations were continued.

At different time intervals during the incubations samples of 20 µl were taken; the reaction was stopped by immediate chilling on ice and addition of 0.98 ml ice-cold $2.5 \times 10^{-2}$ M Tris-HCl pH 8.

DNA inactivation was measured by transfection to *E. coli* spheroplasts, as described in detail elsewhere (van Maanen *et al.*, 1988*b*). $T_{37}$ values, i.e. incubation times resulting in 63% DNA inactivation, were calculated by plotting the surviving fraction of DNA semi-logarithmically versus incubation time by means of a least squares fit.

### Results

### *Electron spin resonance spectroscopy (ESR)*

Upon incubation of the ortho-quinone of etoposide in $5 \times 10^{-2}$ M potassium phosphate pH 4 no ESR signal could be detected (Figure 2).

In incubations of the ortho-quinone at pH 7.4 to 9, however, an ESR signal was generated which was characteristic of the primary semi-quinone free radical of etoposide (van Maanen *et al.*, 1988*b*; Figure 2). Its intensity increased by elevating the pH of the incubations from 7.4 to 9; on the other hand, the signal disappeared far more rapidly at the higher pH values (Figure 3). At pH 10 both the intensity and the rate of disappearance of the signal were essentially similar as at pH 9.

At still higher pH values (>10) an additional ESR signal was detected (data not shown), which could be ascribed to a secondary semi-quinone free radical, possibly derived from the 3′, 4′, 6′-trihydroxy-derivative of etoposide (van Maanen *et al.*, 1988*a*). After about 30 min at pH 12.5 only the latter signal was still detectable.

### *High performance liquid chromatography (HPLC)*

When the ortho-quinone of etoposide was incubated in $5 \times 10^{-2}$ M potassium phosphate pH 4 for increasing periods of time, no changes in the HPLC pattern were observed until at least 5 days (Figure 4a).

In incubations at pH 7.4 the ortho-quinone disappeared in 2–3 h of incubation (Figure 5a); this was accompanied by the formation of more polar conversion products, but also by the appearance of a compound with the same retention time as that of synthetically prepared catechol of etoposide (Figures 4b and 5a). Just as for catechol, this product could be electrochemically oxidised at + 200 mV to a compound which co-eluted with the ortho-quinone (data not shown). We therefore concluded that we were dealing with the catechol of etoposide in our incubation mixtures with the ortho-quinone at pH 7.4. The amount of catechol formed reached a maximum after 2–3 h of incubation (Figure 5a); it

**Figure 2** ESR signals obtained after incubation of 4.4 × $10^{-4}$ M ortho-quinone of etoposide at 37°C for 10 minutes in 5 × $10^{-2}$ M potassium phosphate pH 4 (**a**), 7.4 (**b**) or 9 (**c**). Instrumental conditions: magnetic field, 3460 G; scan range, 20 G; modulation frequency, 100 kHz; modulation amplitude, 0.197 G; gain, 3.20 × $10^5$; power, 50 mW; conversion time, 10.24 ms; number of scans, 20; scan time, 40 s.

**Figure 3** Intensity (in arbitrary units, a.u.) and duration of the ESR signals from the semi-quinone free radical of etoposide, measured at different time intervals during the incubation of 4.4 × $10^{-4}$ M ortho-quinone at 37°C in 5 × $10^{-2}$ M potassium phosphate pH 7.4 (■——■), 8.6 (◆——◆) or 9 (●——●). Instrumental conditons were the same as described in legend to Figure 2.

**Figure 4** HPLC analyses of the ortho-quinone of etoposide (50 µl; 4.4 × $10^{-4}$ M) after incubation at 37°C in 5 × $10^{-2}$ M potassium phosphate: (**a**) pH 4, $t = 0-5$ days or pH 7.4 or 9, $t = 0$ minutes; (**b**) pH 7.4, $t = 2-3$ h; (**c**) pH 7.4, $t = 24$ h; or (**d**) pH 9, $t = 30$ min. Q, ortho-quinone; C, catechol.

then slowly disappeared with the concomitant formation of more polar conversion products (Figure 4c).

At pH 9 the ortho-quinone disappeared about 5 times faster than at pH 7.4; its decay was accomplished within about 30 min of incubation (Figure 5b). This was again accompanied by an extensive production of compounds with shorter retention times, but in contrast to the incubations at pH 7.4, catechol formation was not observed at pH 9 (Figure 4d).

### *Inactivation of ΦX174 DNA*

Exposure of ss ΦX174 DNA to the ortho-quinone of etoposide at pH 4, 7.4 and 9 resulted in an increased rate of DNA inactivation with increasing pH (Figure 6; Table I). Preincubation of the ortho-quinone at pH 7.4 for 2–3 h, which resulted in extensive catechol production and in a very low rate of semi-quinone formation (Figure 3), led to a diminished rate of DNA inactivation (Table I). The rate of DNA inactivation by the catechol of etoposide, synthesised from the ortho-quinone by reduction with ascorbic acid, was of the same magnitude (Table I). Complete conversion of the ortho-quinone into more polar components by preincubation at pH 9 for 35 min resulted in a drastic decrease in DNA inactivation.

RF ΦX174 DNA was only inactivated in incubations with the ortho-quinone at pH 4, but at a lower rate than ss ΦX174 DNA (Figure 7; Table I).

$T_{37}$ values observed under the different experimental conditions are summarised in Table I.

**Figure 5** Decay of the ortho-quinone of etoposide (50 µl; $4.4 \times 10^{-4}$ M) upon incubation at 37°C in $5 \times 10^{-2}$ M potassium phosphate pH 7.4 (■——■) and formation of the catechol from the ortho-quinone at this pH (▲——▲). **b**, Decay of the ortho-quinone of etoposide (50 µl; $4.4 \times 10^{-4}$ M) upon incubation at 37°C in $5 \times 10^{-2}$ M potassium phosphate pH 9.

**Figure 6** Survival curves of ss ΦX174 DNA incubated with $4.4 \times 10^{-4}$ M ortho-quinone of etoposide at 37°C in $5 \times 10^{-2}$ M potassium phosphate pH 4 (▲——▲), 7.4 (■——■) or 9 (●——●). The survival curve obtained at pH 4 was corrected for the formation of apurinic sites due to the acidic incubation mixture alone (correction factor of about 40%). The incubation mixtures of pH 7.4 and 9 did not affect the biological activity of ss ΦX174 DNA significantly during the time course of the measurements.

**Table I** $T_{37}$ values[a] for inactivation of ss or RF ΦX174 DNA (125 ng $ml^{-1}$) by the ortho-quinone (Q) or catechol (C) of etoposide ($4.4 \times 10^{-4}$ M for ss ΦX174 DNA, $1.8 \times 10^{-3}$ M for RF ΦX174 DNA) and the conversion products of the ortho-quinone upon incubation at 37°C under different experimental conditions

| | | | |
|---|---|---|---|
| *ss ΦX174 DNA* | Q, pH 4[b] | | 28±4 min |
| | Q, pH 7.4[c], | 0–2 h | 8±2 min |
| | | 2–3 h preincub. | 90±4 min |
| | C, pH 7.4[c] | | 98±5 min |
| | Q, pH 9[c] | | 2±1 min |
| | | 35 min preincub. | n.s.e.[e] |
| *RF ΦX174 DNA* | Q, pH 4[d] | | 112±6 min |
| | Q, pH 7.4[d] | | n.s.e.[e] |
| | Q, pH 9[d] | | n.s.e.[e] |

[a]Results of at least two experiments. [b]The $T_{37}$value for ss ΦX174 DNA inactivation by the ortho-quinone at pH 4 was corrected for the formation of apurinic sites due to the acidic incubation mixture alone (correction factor of about 40%). [c]The biological activity of ss ΦX174 DNA was not significantly affected by the incubation mixtures of pH 7.4 and 9 alone. $T_{37}$ values were calculated from the initial slopes of the survival curves. [d]The biological activity of RF ΦX174 DNA was not significantly affected by none of the pH values applied. [e]n.s.e. = no significant effect.

**Figure 7** Survival curves of RF ΦX174 DNA incubated with $4.4 \times 10^{-4}$ M ortho-quinone of etoposide at 37°C in $5 \times 10^{-2}$ M potassium phosphate pH 4 (▲——▲), 7.4 (■——■) or 9 (●——●). None of the incubation mixtures affected the biological activity of RF ΦX174 DNA significantly during the time course of the measurements.

### Discussion

Several reports on quinoid anticancer agents suggest an involvement of semi-quinone free radicals in their mechanisms of action (Powis, 1987). In the experiments presented in this paper the DNA inactivating potential of the semi-quinone free radical, produced from the ortho-quinone of etoposide, was investigated. The ESR measurements showed no formation of radical species from the ortho-quinone at pH 4, but an increasing formation of the primary semi-quinone free radical upon elevating the pH from 7.4 to 9 (Figure 2). At pH values ⩾ 11 a secondary semi-quinone free radical was generated. The durations of the ESR signals were inversely related to their intensities (Figure 3). The chemical stability of the ortho-quinone at pH 4 was confirmed by the HPLC experiments, which further demonstrated at pH 7.4 and 9 the conversion of the ortho-quinone into more polar components and at pH 7.4 the formation of also the catechol of etoposide (Figure 4).

A tentative scheme which could explain these observations is depicted in Figure 8. This scheme is derived from previous data on the formation of semi-quinone free radicals from structurally related ortho-quinoid compounds (Ashworth & Dixon, 1972; Dryhurst *et al.*, 1982; Stone & Waters, 1965; Swartz, 1984; van Maanen *et al.*, 1988*b*). Based on these data it can be suggested that ortho-quinones, which are chemically

**Figure 8** Possible conversions of the ortho-quinone of etoposide in potassium phosphate pH ≥ 7.4, leading to the formation of the semi-quinone free radical, the catechol and other non-radical conversion products. (**a**) ortho-qiunone; (**b**) 3′, 4′, 6′-trihydroxy-derivative; (**c**) para-quinone; (**d**) 3′,4′-dihydroxy-derivative (catechol); (**e**) primary semi-quinone free radical (**f**) secondary semi-quinone free radical.

stable at pH 4, become instable at alkaline pH values due to nucleophilic attack by hydroxyl ions at the least protected C-6′ position (reaction 1). The resulting 3′, 4′, 6′-trihydroxy-derivative can be oxidized by the ortho-quinone, yielding a para-quinone, while reducing the ortho-quinone to a catechol (reaction 2). A primary semi-quinone free radical can be produced both in a comproportionation reaction between the ortho-quinone and the catechol (reaction 3) and by oxidation of the latter compound (reaction 4). Oxidation of the 3′, 4′, 6′-trihydroxy-derivative may lead to the formation of a secondary semi-quinone free radical (reaction 5). Disproportionation of primary semi-quinone free radicals (reaction 6) and recombination of primary and secondary semi-quinone free radicals (reaction 7) may reproduce the ortho-quinone and the catechol on the one hand and the para-quinone and the catechol on the other hand. These compounds can take part again in reactions (1) to (5), thus promoting semi-quinone formation. Since the rate of especially reaction (1) is strongly pH-dependent, a faster conversion of the ortho-quinone into semi-quinone free radical species can be expected with increasing alkaline pH.

These data may explain the results from the present ESR and HPLC measurements with the ortho-quinone of etoposide. The increasing formation of the primary semi-quinone free radical (Figures 2 and 3) and the faster decay of the ortho-quinone (Figures 4 and 5) upon elevating the pH of the incubations from 7.4 to 9 can be explained by the higher rates of reactions (1) to (5) with increasing $OH^-$ excess. At still higher pH values reactions (1) and (5) will be shifted strongly to the right; this can increase the rate of formation of the 3′, 4′, 6′-trihydroxy-derivative, which may account for the detection of the secondary semi-quinone free radical at pH values ⩾ 11. Since also reactions (6) and (7) are shifted more to the right with a greater availability of primary and secondary semi-quinone free radicals, the decreasing stability of the two radical species with increasing pH can be explained. At higher pH values the catechol is converted faster into the semi-quinone free radical (reaction 4; van Maanen *et al.*, 1988*a*; Kalyanaraman *et al.*, 1989). Moreover, under these conditions the rate of the comproportionation reaction between the ortho-quinone and the catechol (reaction 3) will be accelerated. The presence of the catechol in the incubations at pH 7.4, but its absence in the incubations at pH 9 (Figure 4) can thus be explained. The appearance of products with shorter retention times in the incubations of the ortho-quinone at pH 7.4 and 9 (Figure 4b–d) may be attributed to the formation of the more polar 3′, 4′, 6′-trihydroxy- and para-quinone-derivatives and possibly also to further degradation of these compounds.

From the chemical stability of the ortho-quinone of etoposide in potassium phosphate pH 4 and from the absence of an ESR signal under these conditions, it can be concluded that the inactivation of ss ΦX174 DNA observed at pH 4 (Figure 6; Table I) is due to this compound itself. Elevating the pH of the ortho-quinone incubations to 7.4 and subsequently to 9 resulted in about a 3.5- and 14-fold, respectively, increased rate of inactivation of ss ΦX174 DNA (Figure 6; Table I). Under these incubation conditions the semi-quinone free radical is generated at an increasing rate from the ortho-quinone (Figures 2 and 3), accompanied by the production of the catechol and of more polar conversion products (Figure 4). The more polar conversion products did not inactivate ss ΦX174 DNA significantly, while the catechol inactivated ss ΦX174 DNA at a lower rate than the ortho-quinone itself (Table I). Taken together, these results suggest that it is the semi-quinone free radical which is the main species in the incubation mixtures with the ortho-quinone at pH values ⩾ 7.4, responsible for the inactivation of ss ΦX174.

Alternatively to the semi-quinone free radical, oxygen-derived free radicals could have caused the inactivation of ss ΦX174 DNA. Hydroxyl radicals can be formed from superoxide anions, which can be produced during semi-quinone formation from the ortho-quinone (Powis, 1987). Hydroxyl radicals can also be produced during semi-quinone generation from the catechol, catalysed by traces of iron (Kalyanaraman *et al.*, 1989; Sinha *et al.*, 1988). Experiments in our laboratory with the hydroxyl radical scavengers *t*-butanol and DMSO, the spin trapping agent DMPO, the enzymes catalase and superoxide dismutase, the iron chelator EDTA and the $O_2^{-\cdot}$ and $HO_2^{\cdot}$ generator potassium superoxide, showed, however, that hydroxyl radicals are most probably not involved in the inactivation of ss ΦX174 DNA under our experimental conditions (van Maanen *et al.*, 1990). Superoxide anions, on the other hand, could contribute to the inactivation of ss ΦX174 DNA by promoting the conversion of the ortho-quinone into the semi-quinone free radical (van Maanen *et al.*, 1990).

In contrast to ss ΦX174 DNA, RF ΦX174 DNA was not significantly inactivated in incubations with the ortho-quinone at pH values ⩾ 7.4, but only at pH 4, hence by the ortho-quinone itself (Figure 7; Table I). The lower rate of RF ΦX174 DNA inactivation by the ortho-quinone of etoposide as compared to that of ss ΦX174 DNA inactivation ($T_{37}$ values of 112 *vs* 26 minutes) indicates that the ortho-quinone is more lethal to ss than to RF ΦX174 DNA. This could be explained by the availability of more binding sites for the ortho-quinone on ss DNA (Kalyanaraman *et al.*, 1989) and/or to excision repair, which acts only on double-stranded RF ΦX174 DNA (van Maanen et al., 1988*b*).

The absence of inactivation of RF ΦX174 DNA in incubation mixtures with the ortho-quinone at pH values ⩾ 7.4 suggests, that the semi-quinone free radical of etoposide does not inflict lethal damage to RF ΦX174 DNA. Since the ortho-quinone, on the other hand, is demonstrated to be lethal to both ss and RF ΦX174 DNA, it can be argued that the semi-quinone free radical interacts differently with DNA as compared to the ortho-quinone and produces different types of DNA damage. This assumption is supported by our recent observations, which indicate that the reaction of the ortho-quinone with both ss and RF ΦX174 DNA leads to the formation of mainly lethal adducts, whereas the semi-quinone free radical induces both adducts and alkali-labile sites, which affect the biological activity of only ss ΦX174 DNA, not that of RF ΦX174 DNA.

From all these data it can be concluded that the semi-quinone free radical of etoposide is able to react with DNA. This conclusion is at variance with the suggestion recently made by Kalyanaraman *et al.* (1989), despite the fact that the data from their ESR measurements are completely in line with our results. These investigators demonstrated in the presence of double-stranded calf thymus DNA a reduced rate of formation of the semi-quinone free radical, which is even more pronounced when single-stranded (denatured) DNA is added. These effects have been, however, interpreted not to be due to a reaction of the DNA with the semi-quinone free radical, but with the ortho-quinone, which may be formed in a back-reaction from the radical. This suggestion is mainly based on the observation that addition of ortho-phenylenediamine, which is known to react with ortho-quinones but not with ortho-semi-quinones, shows a similar reducing effect on the rate of formation of the semi-quinone free radical of etoposide as calf thymus DNA (Kalyanaraman *et al.*, 1989). This, of course, does not exclude beforehand the possibility that reactions of this radical species with DNA can take place. In fact, such reactions do take place, as can be concluded from the results of our experiments.

Our finding, that the semi-quinone free radical of etoposide is able to inflict inactivating damage to single-stranded DNA suggests, that this radical species, in addition to the ortho-quinone and the catechol, might also be involved in the cytotoxicity of etoposide. It is generally known that the cellular DNA is subjected to a number of DNA–protein interactions and enzymatic processes, which are accompanied by a temporary and partial DNA strand separation. In particular during DNA replication in the S-phase of the cell cycle, extensive strand separation takes place and the DNA is present in single-stranded form at the many replication forks. If these single-stranded sites are accessible to free radical attack, a considerable inhibition of the DNA replication can be expected. In this way, the semi-quinone free radical of etoposide might contribute to the observed cell cycle delay in the late S- and $G_2$-phases by the parent compound (Loike & Horwitz, 1976; Stähelin, 1973; Smith *et al.*, 1986). Also the earlier suggestions on a mechanism of action of etoposide involving oxidation–reduction processes (Wozniak & Ross, 1983) and free radical species (Wozniak *et al.*, 1984) are in accordance with the present findings.

Taken together, the results presented in this paper, combined with those previously obtained by others (for a recent review see van Maanen *et al.*, 1988*c*), give further support for a mechanism of action of etoposide based on its bioactivation to DNA damaging metabolites, in addition to topoisomerase II inhibition by the drug.

Thanks are due to J. Pluijmackers-Westmijze and R. Hoed (Department of Biophysics, Free University, Amsterdam) for their technical assistance with the transfection experiments. Supported by the Netherlands Cancer Foundation (grant IKA 85–06).

### References

ASHWORTH, P. & DIXON, W.T. (1972). Secondary radicals in the autoxidation of hydroquinones and quinones. *J. Chem. Soc. Perkin*, **II**, 1130.

BAAS, P.D., TEERTSTRA, W.R., VAN MANSVELD, A.D.M. & JANSZ, H.S. (1981). Construction of viable and lethal mutations in the origin of bacteriophage ΦX174 using synthetic oligodeoxyribonucleotides. *J. Mol. Biol.*, **152**, 615.

BLOK, J., LUTHJENS, L.H. & ROOS, A.L.M. (1967). The radiosensitivity of bacteriophage DNA in aqueous solution. *Radiat. Res.*, **30**, 468.

CHEN, G.L., YANG, L., ROWE, T.C., HALLIGAN, B.D., TEWEY, K.M. & LIU, L.F. (1984). Non-intercalative antitumour drugs interfere with the breakage-reunion reaction of mammalian DNA topoisomerase II. *J. Biol. Chem.*, **259**, 13560.

DRYHURST, G., KADISH, K.M., SCHELLER, F. & RENNEBERG, R. (1982). Catecholamines. In *Biological Electrochemistry Vol. 1*, (eds) p. 158. Academic Press: New York.

GLISSON, B.S., SMALLWOOD, S.E. & ROSS, W.E. (1984). Characterization of VP-16-induced DNA damage in isolated nuclei from L1210 cells. *Biochim. Biophys. Acta*, **783**, 74.

HAIM, N., NEMEC, J., ROMAN, J. & SINHA, B.K. (1987). Peroxidase-catalyzed metabolism of etoposide (VP-16-213) and covalent binding of reactive intermediates to cellular macromolecules. *Cancer Res.*, **47**, 5835.

HOLTHUIS, J.J.M., VAN OORT, W.J., RÖMKENS, F.M.G.M., RENEMA, J. & ZUMAN, P. (1985). Electrochemistry of podophyllotoxin derivatives. I. Oxidation mechanism of etoposide (VP-16-213). *J. Electroanal. Chem.*, **184**, 317.

ISSELL, B.F., MUGGIA, F.M. & CARTER, S.K. (eds.) (1984). *Etoposide (VP 16). Current Status and New Developments*. Academic Press: Orlando, FL.

KALYANARAMAN, B., NEMEC, J. & SINHA, B.K. (1989). Characterization of free radicals produced during oxidation of etoposide (VP-16) and its catechol and quinone derivatives. An ESR study. *Biochemistry*, **28**, 4839.

LOIKE, J.D. & HORWITZ, S.B. (1976). Effect of VP-16-213 on the intracellular degradation of DNA in HeLa cells. *Biochemistry*, **15**, 5443.

LONG, B.H., MUSIAL, S.F. & BRATTAIN, M.G. (1984). Comparison of cytotoxicity and DNA breakage activity of congeners of podophllotoxin including VP-16-213 and VM-26: a quantitative structure-activity relationship. *Biochemistry*, **23**, 1183.

MANS, D.R.A., VAN MAANEN, J.M.S., LAFLEUR, M.V.M., VAN SCHAIK, M.A., RETÈL, J. & LANKELMA, J. (1989). Role of the semi-quinone free radical of VP-16-213 in the inactivation of single-stranded ΦX174 DNA. *Proc. Am. Assoc. Cancer Res.*, **30**, 489.

NEMEC, J., FINCH, R.A., AVERY, T.L. (1985). Epipodophyllotoxin-quinone derivatives – new etoposide and teniposide analogues. *Proc. Am. Assoc. Cancer Res.*, **26**, 258.

POWIS, G. (1987). Metabolism and reactions of quinoid anticancer agents. *Pharmac. Ther.*, **35**, 57.

ROSS, W.E., ROWE, T., GLISSON, B.S., YALOWICH, J. & LIU, L. (1984). Role of topoisomerase II in mediating epipodophyllotoxin-induced DNA cleavage. *Cancer Res.*, **44**, 5857.

SINHA, B.K. & MYERS, C.E. (1984). Irreversible binding of etoposide (VP-16-213) to deoxyribonucleic acid and proteins. *Biochem. Pharmacol.*, **33**, 3725.

SINHA, B.K., ELLIOT, H.M. & KALYANARAMAN, B. (1988). Iron-dependent hydroxyl radical formation and DNA damage from a novel metabolite of the clinically active antitumor drug VP-16. *FEBS Lett.*, **227**, 240.

SMITH, P.J., ANDERSON, C.O. & WATSON, J.V. (1986). Predominant role for DNA damage in etoposide-induced cytotoxicity and cell cycle perturbation in human SV40-transformed fibroblasts. *Cancer Res.*, **46**, 5641.

STÄHELIN, H. (1973). Activity of a new glycosidic lignan derivative (VP-16-213) related to podophyllotoxin in experimental tumors. *Eur. J. Cancer*, **9**, 215.

STONE, T.J. & WATERS, W.A. (1965). Aryloxy-radicals. Part IV. Electron spin resonance spectra of some ortho-monobenzosemiquinones and secondary radicals derived therefrom. *J. Chem. Soc.*, 1488.

SWARTZ, H.M. (1984). Electron spin resonance studies of cancer: experimental results and conceptual implications. In *Free Radicals in Molecular Biology, Aging and Disease*, Armstrong, D. *et al.* (eds) p. 275. Raven Press: New York.

VAN MAANEN, J.M.S., DE RUITER, C., KOOTSTRA, P.R. & 4 others (1985). Inactivation of ΦX174 DNA by the ortho-quinone derivative or its reduction product of the antitumor agent VP-16-213. *Eur. J. Cancer Clin. Oncol.*, **21**, 1215.

VAN MAANEN, J.M.S., DE VRIES, J., PAPPIE, D. & 5 others (1987). Cytochrome P-450-mediated O-demethylation: a route in the metabolic activation of etoposide. *Cancer Res.*, **47**, 4658.

VAN MAANEN, J.M.S., VERKERK, U.H., BROERSEN, J. & 4 others (1988*a*). Semi-quinone formation from the catechol and ortho-quinone metabolites of the antitumor agent VP-16-213. *Free Rad. Res. Commun.*, **6**, 371.

VAN MAANEN, J.M.S., LAFLEUR, M.V.M., MANS, D.R.A. & 7 others (1988*b*). Effects of the ortho-quinone and catechol of the antitumor drug VP-16-213 on the biological activity of single-stranded and double-stranded ΦX174 DNA. *Biochem. Pharmacol.*, **37**, 3579.

VAN MAANEN, J.M.S., RETÈL, J., DE VRIES., J. & PINEDO, H.M. (1988*c*). Mechanism of action of antitumor drug etoposide: a review. *J. Natl Cancer Inst.*, **80**, 1526.

VAN MAANEN, J.M.S., MANS, D.R.A., LAFLEUR, M.V.M. & 4 others (1990): Effects of oxygen radical scavengers on the inactivation of single-stranded ΦX174 DNA by the semi-quinone free radical of the antitumor agent etoposide *Free Rad. Res. Commun.* (in the press).

WOZNIAK, A.J. & ROSS, W.E. (1983). DNA damage as a basis for 4′-demethylepipdophyllotoxin-9- (4,6-0-ethylidene-β-D-glucopyranoside) (etoposide) cytotoxicity. *Cancer Res.*, **43**, 120.

WOZNIAK, A.J., GLISSON, B.S., HANDE, K.R. & ROSS, W.E. (1984). Inhibition of etoposide-induced DNA damage and cytotoxicty in L1210 cells by dehydrogenase inhibitors and other agents. *Cancer Res.*, **44**, 626.

YALOWICH, J.C. & ROSS, W.E. (1984). Potentiation of etoposide-induced DNA damage by calcium antagonists in L1210 cells *in vitro*. *Cancer Res.*, **44**, 3360.